# Effect of adding systematic desensitization to goal-directed paradigm on risk of falling in patients with stroke: a randomized controlled trial

**DOI:** 10.3389/fneur.2024.1285420

**Published:** 2024-05-09

**Authors:** Heba Mohammed Gaber Abdelfadil, Ebtisam Mohamed Fahmy, Shimaa Mohamed Abdelmegeed, Hoda Mohammed Zakaria, Ashraf Ahmed Darwesh, Ahmed Mahmoud Kadry, Shereen Hamed Elsayed, Ahmed M. Aboeleneen, Ahmed Magdy Alshimy

**Affiliations:** ^1^Department of Physical Therapy for Neuromuscular Disorders and its Surgery, Faculty of Physical Therapy, October 6 University, Giza, Egypt; ^2^Department of Neurology, Faculty of Medicine, Cairo University, Giza, Egypt; ^3^Department of Physical Therapy for Neurology and Neurosurgery, Faculty of Physical Therapy, Cairo University, Giza, Egypt; ^4^Faculty of Physical Therapy, Kafrelsheikh University, Kafr El Sheikh, Egypt; ^5^Department of Rehabilitation Sciences, College of Health and Rehabilitation Sciences, Princess Nourah bint Abdulrahman University, Riyadh, Saudi Arabia; ^6^Department of Physical Therapy, Faculty of Medical Rehabilitation Sciences, King Abdulaziz University, Jeddah, Saudi Arabia; ^7^Department of Basic Science, Faculty of Physical Therapy, Cairo University, Giza, Egypt; ^8^Department of Physical Therapy for Neurology and Its Surgery, Faculty of Physical Therapy, Al Ryada University for Science and Technology, Sadat City, Menoufia, Egypt

**Keywords:** stroke, risk of falling, systematic desensitization, balance, goal-directed paradigm

## Abstract

**Background:**

Improvement in rehabilitation outcomes for patients suffering from chronic stroke can be attained through systematic desensitization of their fear of falling, which in turn reduces the risk of falling.

**Purpose:**

This study aimed to examine the effect of adding systematic desensitization to a goal-directed paradigm on functional performance, balance, risk of falling, and fear of falling among chronic ischemic stroke patients.

**Methodology:**

Two equally sized groups, each comprising 40 stroke patients of both sexes, were randomly divided. For 8 weeks, Group A received three sessions per week of combined treatment consisting of systematic desensitization and a goal-directed paradigm, while Group B received only the goal-directed paradigm. The Timed Up and Go (TUG) test and Dynamic Gait Index (DGI) were used to assess function performance; the Berg Balance Scale (BBS) and the Biodex Fall Risk Index (FRI) were used to evaluate balance; and the 16-item Fall Efficacy Scale-International (FES-I) was used to evaluate fear of falling. At baseline and after the treatment, all measurements were obtained.

**Results:**

Both groups (A and B) revealed a substantial increase in functional performance through a decrease in TUG scores and an increase in DGI. Additionally, there was a decrease in the risk of falling through an increase in the BBS scores and a decrease in the FRI. Furthermore, there was a decrease in the fear of falling, as measured using the FES-I, after treatment, with superior improvement in Group A with a *p*-value of <0.001.

**Conclusion:**

Systematic desensitization combined with a goal-directed paradigm has a superior effect on improving functional performance and reducing the risk of falling and the fear of falling in patients with stroke compared to a goal-directed paradigm alone.

## 1 Introduction

Stroke patients may have difficulties maintaining their balance due to various factors, including injury to the brain or systems responsible for maintaining balance, weakness of the muscles, loss of sensation, visual impairments, and dizziness. Impaired physical and mental functions can contribute to falling after a stroke (1).

Stroke patients often experience cognitive disorders, depression, anxiety, low self-confidence, and difficulties in carrying out activities of daily living (ADLs), in addition to paralysis of their limbs. They are more prone to falling because of weakened lower limbs, gait impairment, decreased postural stability, and lowered attention (2).

Fear of falling (FoF) is a psychiatric problem linked to post-stroke fall risks and balance issues. It might prevent an individual from engaging in everyday activities and maintaining their physically activity. The fear of falling, or “basophobia,” can be experienced by individuals who have never had a fall before, and it can contribute to future falls or injury (3).

In stroke populations, fear of falling may cause avoidance behaviors, which can worsen disability by triggering inactivity and social isolation. Self-efficacy expectations related to falls are a significant risk factor for falling in stroke patients. Approximately 60%−70% of chronic stroke patients report experiencing FoF, which is linked to elevated anxiety and impaired mobility and balance (4).

Phobic anxiety, which affects 18%−25% of stroke patients and has crippling effects on the quality of life, everyday functioning, and social contacts, is another common severe complication after stroke, along with reduced postural control (5).

Systematic desensitization is commonly seen as a behavioral or cognitive-behavioral treatment. It is one of the first forms of exposure therapy to emerge in the era of behavior therapy. Exposure therapy can be further explained as a series of psychological treatment strategies that aims to reduce pathological fears commonly found in people with anxiety disorders. Systematic desensitization is usually considered a form of behavioral or cognitive-behavioral therapy (CBT) (6). Furthermore, CBT techniques could be used in the treatment of chronic pain, cancer, and other severe conditions such as stroke and could help in enhancing physical and mental abilities while reducing undesirable behaviors (7).

Systematic desensitization helps individuals replace negative cognitive, emotional, or behavioral reactions with coping strategies and self-confidence. It is particularly useful for those with balance issues to overcome their fear of falling. This therapy helps people change their negative thoughts, improve their confidence in maintaining balance, and replace their exaggerated fears and unrealistic expectations of falling with a more positive and realistic outlook. This makes them less susceptible to falls and has a high tendency toward fall prevention (8). Systematic desensitization is a successful technique that helps reduce or eliminate the anxious behaviors generated by anxiety-inducing stimuli. These stimuli trigger fear structures, which are networks of links between anxiety-related cognitions, feelings, thoughts, and behaviors such as avoidance or escape. When new information that contradicts the original fear thoughts is obtained, the maladaptive fear linkage is weakened and replaced (9).

Jacobson's progressive muscle relaxation technique was incorporated into systemic desensitization as the main anxiety-inhibiting process. Interactive guided imagery therapy, when used in conjunction with other therapeutic techniques, can be advantageous because it can increase a person's quality of life and capacity for coping. Once the patient has mastered this technique, they can use it almost anytime and in different situations, both inside and outside of the therapist's office (7, 10, 11).

Goal-directed therapy (GDT) is highly customized, client-centered, and focused on function. GDT is consistent with motor development and control concepts such as intense motor training, varying practice, and provision of feedback. Activity-based treatment provides individuals with a variety of opportunities to learn skills that can be used in everyday life (12). In addition, GDT allows the motor cortex to reorganize and promotes neuroplasticity. It has additionally been demonstrated that goal-oriented therapy enhances the ability of a participant to work after a stroke more than traditional therapy. Moreover, this therapy enhances participants' overall quality of life, with results that are much quicker and easier (13). GDT can improve the balance and gait of stroke patients through enhanced activity in the ipsileisonal primary sensory-motor cortex as well as redistributing of activity in various parts of the sensorimotor system. Additionally, GDT improves neuroplasticity associated with intensive practice (14, 15).

Furthermore, GDT can preferentially activate self-efficacy, which is a creative capacity that requires the integration of several types of knowledge and skill research, including intellectual, social, and behavioral capacities (13). Furthermore, verbal encouragement and feedback from the physical therapist during exercise may also promote a sense of balance self-efficacy (16).

Understanding stroke, addressing a patient's fear of falling, and developing comprehensive treatment in this area may help break the cycle of fear and risk of falling, reducing anxiety, promoting community involvement, and improving the quality of life (17). While many other researchers have tested the effect of GDT on the risk of falling (18–21) and fear of falling (16), as well as the effect of CBT on confidence and fear of falling (22, 23) in stroke patients, there remains a lack of primary research that tests the effect of a desensitization approach, as a CBT intervention, in addition to GDT, to tackle this problem. Therefore, this study was conducted to investigate whether adding systematic desensitization to the goal-directed paradigm would be better than GDT alone in improving functional mobility and balance as well as reducing the risk of falling and the fear of falling in patients with stroke.

## 2 Methods

### 2.1 Design

This study was a multicenter randomized controlled trial, with a 1:1 allocation ratio and single-blinded assessor. The study protocol has been approved by the physical therapy research ethical committee, Cairo University (P.T.REC/012/003940), and was clinically registered with clinical trial registration number: NCT05581537. Before being enrolled in the study, all patients signed a written consent form.

### 2.2 Participants

The research involved a multicenter design and included 40 patients of both sexes, aged between 45 and 60 years, who experienced chronic ischemic stroke. These patients were referred by a neurologist to either the outpatient clinics at the 6th of October University Hospital or the Faculty of Physical Therapy, located in Cairo, Egypt. The study was conducted from July 2022 to August 2023.

Patients were eligible for the study if they had experienced an ischemic stroke more than 3 months prior, had a history of falling according to their neurologist referral form, had adequate cognition (as measured by a Montreal Cognitive Assessment score of over 26), and exhibited spasticity in the upper and lower extremities ranging from grade 1+ and 2 on a modified Ashworth scale.

Patients were excluded from the trial if they had musculoskeletal difficulties such as severe arthritis, complete hip arthroplasty, knee surgery, lower limb fractures during the past 6 months, or contractures caused by a fixed deformity and/or leg length discrepancy. Unstable medical issues (e.g., history of congestive heart failure, recent myocardial infarction, substantial cardiac valve problems, unstable angina, or unstable hypertension), speech, hearing, and visual impairments, and epilepsy or psychiatric disorders were also considered as the exclusion factors.

### 2.3 Interventions

Forty patients participating in the study were randomly assigned to two equally sized groups: Study Group (Group A) and Control Group (Group B). Group A received a combined treatment of systematic desensitization for 50 min and a goal-directed paradigm for 40 min, while Group B only received a goal-directed paradigm for 40 min. The same physical therapist delivered the intervention at both sites. Therapy sessions for both groups were conducted three times a week for 8 weeks at the location where they were recruited.

#### 2.3.1 Systematic desensitization intervention

Patients in the study group performed two techniques of systemic desensitization (progressive muscle relaxation and guided imagery training) before starting their goal-directed paradigm to bring the patient to mental relaxation, aiming to reduce the psychological fear that may lead to an actual fall and reduce the physical exertion while performing the motor tasks.

##### 2.3.1.1 Jacobson's technique (progressive muscle relaxation)

This technique involves tightening flow by relaxing the muscles as the patient moves through the body and concentrating on mental relaxation as the patient performs the exercise (10). The duration of progressive muscle relaxation was about 20 min. The duration of each muscle tension was (7–10 s), and the duration of muscle relaxation was (15-20 s) (11).

##### 2.3.1.2 Guided imagery training

It is a relaxation technique that establishes the connection between the brain, mind, body, and behavior. Relaxing means being free from physical, emotional, and mental stress. In this therapy method, the patient replaces negative or stressful thoughts with positive ones. It is a treatment used to relieve pain and anxiety while increasing relaxation. In our study, the patient was motivated to imagine a picture that might assist in reducing agony. Interactive guided imagery therapy was conducted for 30 min (24).

##### 2.3.1.3 Goal-directed paradigm (selected program) intervention

The program included the following components: (1) Stretching exercises targeting the hip adductors and external rotators. (2) Facilitation techniques for transitioning from sitting to standing (initially from a chair and then from a Swiss ball). (3) Weight shifting exercises (moving from sitting on a Swiss ball to standing on a firm surface and then on a foam surface). (4) Reaching exercises performed in different planes (transitioning from sitting to standing on a firm surface and then on a foam surface), targeting both the affected and non-affected sides. (5) Squatting exercises with a single leg stance (with holding the ball and then without holding the ball). (6) Gait training (including walking up and down stairs, sideways, forward, backward, and pivot walking).

### 2.4 Outcome measures

All patients were evaluated using the following assessments before and after treatment.

#### 2.4.1 Functional performance assessment

The functional mobility of the study sample was assessed using the Timed Up and Go (TUG) test. Upon receiving the signal, the patient rises from the chair, walks 3 m to a cone, then walks back to the chair, and sits down. The time it took for the patient from the start of the signal to sitting back in the chair was calculated in seconds (25).

The patient's capacity to sustain walking balance while adapting to different tasks was evaluated using the Dynamic Gait Index (DGI). The patients were asked to perform eight common gait activities, with each item assessed on a four-level ordinal scale. Grade three denotes normal performance, grade two denotes minimum impairment, grade one denotes moderate impairment, and grade zero denotes severe impairment with a maximum score of 24; scores of 19/24 are predictive of a risk of falling in the elderly, and scores >22/24 are indicative of individuals who are safe ambulators (26).

#### 2.4.2 Risk of falling and balance assessment

The Berg Balance Scale was used for assessing patients' balance abilities and fall risk. It is one of the widely used, valid, and reliable tools for the assessment of static and dynamic balance. It consists of 14 mobility tasks, with the tasks varying in degrees of difficulty. Each task is scored on a five-point scale, ranging from 0 to 4. “0” was given at the least level of function, while grade “4” was given at the highest level of function. The scores were classified as follows: 41–56 = low fall risk, 21–40 = medium fall risk, and 0–20 = high fall risk (27, 28).

The Biodex Balance System, developed by Biodex Medical Systems Inc. in Shirley, NY, evaluates a person's capability to sustain dynamic stability of posture under dynamic stress to measure and train neuromuscular control. In our study, the Biodex Balance System was used to assess the risk of falling by measuring the overall stability index in degrees. This was expressed as the Biodex Fall Risk Index (FRI), following standard procedures. After a detailed explanation of the test and removing their footwear, patients stood on the Biodex Balance platform with their eyes open. At the same time, the Biodex balance device was adjusted to the most stable support surface (level 8). Then, while looking at a vertical screen positioned 30 cm away from them, the patients were asked to remain in a vertical position, placing their center of gravity in the middle of the platform. The fall risk assessment test was performed three times, with each test lasting 20 s and spaced at 10 s intervals. The mean FRI of the three tests was calculated. The test was performed pre- and post-treatment with lower values indicating a better balance status (29).

#### 2.4.3 Fear of falling assessment

The 16-item Fall Efficacy Scale-International (FES-I) was utilized to evaluate the fear of falling using 16 questions with answers varying from one (not concerned) to four (very concerned). For data analysis, the overall score which was the sum of the 16 answers and ranged from 16 to 64 points, was utilized. Patients who had higher overall scores were more anxious about falling (30, 31).

### 2.5 Sample size

In this study, using the G^*^POWER statistical program (version 3.1.9.2; Franz Faul, Universidad Kiel, Germany), the sample size was calculated to detect a 15% reduction in the TUG test as a primary outcome measure. Based on the data obtained from a pilot study, an effect size of 0.91 was determined. To achieve 80% statistical power and 5% significance level, 40 participants (20 in each group) were required for the given effect size.

### 2.6 Randomization and blinding

The selected patients were randomly assigned to Group A (study) and Group B (control) using opaque sealed envelopes. An independent statistician used a computer to create a randomization list for patient allocation. To ensure sex balance, the randomization was stratified by sex. The randomization list was kept confidential and sealed until a patient had provided informed consent and was officially enrolled in the study. To eliminate potential bias, all patient examinations and data collection were conducted by a single trained assessor who was blinded to the patient allocation.

### 2.7 Statistical analysis

The data analysis was conducted using IBM SPSS, version 25 for Windows, in Chicago, IL, USA. An initial alpha level of 0.05 was set. *T*-tests were used to compare numerical data, and chi-squared tests were used to compare nominal data of patients in both groups. Shapiro–Wilk and Levene's tests were performed to check data for normality and homogeneity of variance, and no violations of the parametric assumptions were detected. To examine the effects of treatment (between groups), time (before and after treatment), and their interaction on outcome measures, mixed ANOVA was applied. *Post hoc* tests were conducted to determine significant differences between groups and time points.

## 3 Results

All the recruited patients were all available in the follow-up and final analysis with no dropouts. At the outset, the two groups showed no noticeable differences in terms of age, weight, height, BMI, or illness duration (*p*-value>0.05), as demonstrated in Table 1. Moreover, the study groups had an equal distribution of sex and hemiplegic side, as indicated in Table 2. Additionally, the measured dependent variables (DGI, TUG, BBS, FRI, and FES-I) before treatment showed no statistically significant difference across the groups, with respective *p*-values of 0.384, 0.148, 0.143, 0.402, and 0.452 (Table 3).

**Table 1:** Demographic characteristics of patients in both groups.

	**Group (A) (*n* = 20) mean ±SD**	**Group (B) (*n* = 20) mean ±SD**	***p*-value**	**Sig**.
Age (years)	51.10 ± 5.17	52.25 ± 3.58	0.916	NS
Height (cm)	175.10 ± 8.41	171.80 ± 6.23	0.167	NS
Weight (kg)	79.10 ± 7.66	75.85 ± 4.60	0.112	NS
BMI (Kg/m^2^)	25.76 ± 1.07	25.42 ± 0.81	0.269	NS
Duration of illness (months)	16.90 ± 8.11	18.45 ± 7.68	0.539	NS

p-value, probability value; SD, standard deviation; NS, non-significant.

**Table 2:** The frequency and chi-squared test for comparison of sex and affected side distribution in both groups.

**Group**	**Sex**			
	**Male no (%)**	**Female no (%)**	***p*** **-value**	**Sig**.
Group (A) (*n* = 20)	13 (65 %)	7 (35 %)	0.490	NS
Group (B) (*n* = 20)	15 (75 %)	5 (25 %)		
	**Affected side**			
	**Right no (%)**	**Left no (%)**	***p*** **-value**	**Sig**.
Group (A) (*n* = 20)	8 (40 %)	12 (60 %)	0.342	NS
Group (B) (*n* = 20)	11 (55 %)	9 (45 %)		

χ^2^, Chi-squared value; p-value, probability value; NS, non-significant.

**Table 3:** Descriptive statistics and within and between groups comparison of measured dependent variables.

	**Pre (mean ±SD)**	**Post (mean ±SD)**	***p*-value^α^**
**TUG in seconds**			
Group (A) (*n* = 20)	35.05 ± 8.16	22.50 ± 8.07	<0.001^*^
Group (B) (*n* = 20)	37.00 ± 5.59	31.20 ± 4.91	<0.001^*^
*p*-value^β^	0.384	<0.001^*^	
**DGI**			
Group (A) (*n* = 20)	8.60 ± 1.46	18.80 ± 2.01	<0.001^*^
Group (B) (*n* = 20)	7.95 ± 1.31	10.80 ± 1.05	<0.001^*^
*p*- value^β^	0.148	<0.001^*^	
**BBS score**			
Group (A) (*n* = 20)	34.85 ± 1.87	51.90 ± 4.58	<0.001^*^
Group (B) (*n* = 20)	33.95 ± 1.93	37.85 ± 2.36	<0.001^*^
*p*-value^β^	0.143	0.042^*^	
**FRI**			
Group (A) (*n* = 20)	2.96 ± 0.41	1.50 ± 0.42	<0.001^*^
Group (B) (*n* = 20)	2.86 ± 0.29	2.16 ± 0.55	<0.001^*^
*p*-value^β^	0.402	<0.001^*^	
**FES-I**			
Group (A) (*n* = 20)	52.60 ± 1.84	37.50 ± 1.60	<0.001^*^
Group (B) (*n* = 20)	52.15 ± 1.89	42.25 ± 2.67	<0.001^*^
*p*-value^β^	0.452	<0.001^*^	

TUG, timed up and go test; DGI, Dynamic Gait Index; BBS, Berg Balance Scale; FRI, Biodex Fall Risk Index; FES-I, the 16-item Fall Efficacy Scale-International; MD, Mean difference; α, within group comparison; β, between groups comparison.

^*^Significant at p < 0.05.

To determine how therapy affected the measured dependent variables, mixed ANOVA was utilized. The time and treatment variables exhibited a significant interaction effect (*F* = 122.57, *p*-value <0.001). The treatment main effect (group effect) was significant (*F* = 23.36, *p*-value <0.001). In addition, a significant effect of the time factor was represented (*F* = 1019.34, *p*-value <0.001).

The comparison within groups after treatment revealed that both groups A and B showed a significant improvement in functional mobility as measured by TUG and DGI. The TUG scores decreased significantly (*p*-value <0.001), with Group A improving by 35.80% and Group B by 15.67%. Similarly, the DGI scores increased substantially (*p* <0.001), with Group A improving by 118.60% and Group B by 35.85% (Table 3).

In terms of risk of falling and balance, measured using the BBS and FRI respectively, there was a significant increase in BBS scores (*p* <0.001) in both groups, with an improvement of 48.92% in Group A and 11.48% in Group B. The FRI scores decreased significantly (*p* <0.001), with Group A improving by 49.32% and Group B by 24.47%.

Finally, the fear of falling, measured using FES-I, decreased significantly (*p* <0.001) in both groups. Group A showed an improvement of 28.70%, while Group B improved by 18.98%.

After-treatment results were compared between groups, revealing statistically significant differences with better outcomes in Group A (*p*-value <0.05; Table 3).

## 4 Discussion

The current study investigated the effect of adding systematic desensitization to GDT on improving functional mobility, balance, and reducing fear of falling in ischemic stroke patients. The results showed a significant reduction in the risk of falling that was supported by the significant improvement of functional mobility measured by TUG and DGI, risk of falling and balance measured by BBS and FRI, and a decrease in the fear of falling measured by FES-I in both groups. Furthermore, our study showed a superior result in the study group (A) which received a systematic desensitization and a goal-directed paradigm over the control group (B) which received a goal-directed paradigm only. A statistically significant increase was observed in both groups according to the BBS data. However, the mean values of BBS post-treatment were within the medium fall risk category in Group B (37.85 ± 2.36) and the low fall risk category in Group A (51.90 ± 4.58; Table 3). This indicates more clinical improvement in the risk of falling in Group A.

These outcomes were reported in accordance with previous studies that found that combined treatment of CBT and conventional task-oriented physical therapy improves standing balance and mobility, promotes physical activities, enhances balance, and lowers fear of falling among stroke patients (22, 32, 33). Additionally, attention distraction by an external factor or cognitive tasks significantly reduces postural instability and ankle muscle co-contraction in stroke survivors, especially those with high anxiety, in comparison to healthy subjects (34).

One possible explanation for the positive impact of systematic desensitization on physical function is that it reduces hypertonicity, contractile hypertension in voluntary muscles, approximation of involved muscles, and the disparity of extrafusal-intrafusal muscle fibers associated with it. This facilitates easier movements (32). Furthermore, progressive muscle relaxation could reduce the risk of falling by decreasing physiological tension by repeatedly tensing and relaxing muscles, which in turn reduces the heart rate and blood pressure. It also reduces stress and anxiety by activating the parasympathetic nervous system, which lowers the hypothalamic impulse pressure via focused thought practice that reinforces positive attitudes (35, 36). According to the emotional processing hypothesis, the reduction in fear of falling can be explained by the acquisition of new stimuli that contradict the original fear thoughts. This leads to weakening and replacement of the maladaptive fear linkage (9).

The application of GDT only (control group) in this study has a positive effect on functional performance and balance, which is consistent with previous research on stroke patients. Previous studies have found that training tailored to the specific task (GDT), based on research from rehabilitation science, motor learning, and motor control, is more effective in improving the functionality and the overall quality of life for post-stroke patients compared to traditional treatments (13, 18–21)].

Regarding the significantly improving effect of applying GDT only in the control group, the patient gained more self-assurance and fall efficacy as a result. This is supported by the results of a recent systematic review that found a substantial overall positive effect of GDT on FoF from six studies included in their systemic review analysis (four showed a significant difference between GDT and control) (16). There was no clear explanation for this decrease in FoF after the GDT application. However, according to the theory proposed, exercise may reduce the fear of falling by exposing the patient to a successful non-fall experience while performing several motor tasks (37).

This study presented a superior result for the combination of systemic desensitization with GDT over the use of GDT alone. These results are supported by the work of Liu et al., who tested other types of CBT, specifically cognitive restructuring and behavioral modification, when combined with task-oriented balance training (TOBT). They compared these interventions to TOBT alone and general health education in stroke patients. Combining CBT and TOBT resulted in better subjective balance confidence (22) as well as improvements in daily living activities, fear of falling, balance, and fear avoidance behaviors (33). Similarly, compared to conventional physical therapy alone, adding cognitive training to balance training and conventional physical therapy produced better results in reducing the fear of falling and improving balance (38). Other studies have also supported this approach and have used varying treatment times. For instance, one study found that adding 60 min of CBT to conventional therapy resulted in better outcomes for patients with chronic low back pain, particularly in terms of reducing disability and fear avoidance behaviors (39).

However, our research suggests that the greater improvement observed in the study group was due to the synergistic impact of GDT and systemic desensitization, rather than simply the duration of the therapy session. This is supported by a recent systematic analysis of 21 trials, which found that longer periods of the same type of rehabilitation following a stroke had little to no effect on significant tasks such as daily living and upper and lower limb activities. This means the improvement is more related to the quality and the type of the treatment method than the time of treatment (40). On the other hand, research comparing the effect of physical and cognitive behavioral treatments in chronic low back pain showed that active therapy alone has better results than adding more time of CBT to active physical therapy alone on pain cognition, disability, and depression in chronic low back pain patients (41).

## 5 Strengths and limitations

In our study, we used a variety of objective outcome measures, such as the Biodex System. Additionally, functional outcomes such as DGI, and TUG were used, which are more related to fall risk than static balance parameters (42). However, performing many tests might be difficult to endure by participants, raising the dropout chance. This risk has been mitigated through motivation and explanation of the potential benefits to the patients and their families and by providing them feedback and recognition after each assessment. The findings of the study are generalizable to chronic ischemic stroke patients who have a moderate to high risk of falling and/or fear of falling. However, it should be noted that the study sample mostly consisted of male patients aged between 45 and 60 years from the two centers mentioned. The intervention was standardized and delivered by trained therapists, and the outcome measures were reliable. Further studies are required to replicate the results in different populations and contexts. The available literature suggests that the efficacy of combined therapeutic interventions is primarily determined by their quality and type, rather than the duration of treatment (40). Despite the benefits of the combination of systematic desensitization with GDT, more studies are needed to fully comprehend the impact of different methods and approaches of systematic desensitization, such as the effects of different treatment durations, optimal CBT timing, the comparison of single vs. multiple sessions, and the advantages and disadvantages of individual vs. group therapy. Finally, other studies are recommended to test the long-term effects after the intervention.

## 6 Conclusion

Systematic desensitization combined with a goal-directed paradigm has a superior effect on improving functional performance and balance, reducing the risk of falling, and the fear of falling in patients with ischemic stroke in comparison to a goal-directed paradigm only.

## Data availability statement

The raw data supporting the conclusions of this article will be made available by the authors, without undue reservation.

## Ethics statement

The studies involving humans were approved by Physical therapy research Ethical Committee, Cairo University (approval no. P.T.REC/012/003940). The studies were conducted in accordance with the local legislation and institutional requirements. The participants provided their written informed consent to participate in this study.

## Author contributions

HA: Conceptualization, Investigation, Methodology, Project administration, Writing—original draft, Writing— review & editing. EF: Conceptualization, Formal analysis, Supervision, Validation, Writing—review & editing. SA: Conceptualization, Data curation, Supervision, Writing—original draft, Writing—review & editing. HZ: Conceptualization, Data curation, Formal analysis, Methodology, Supervision, Writing—review & editing. AD: Conceptualization, Formal analysis, Methodology, Supervision, Writing—original draft, Writing—review & editing. AK: Data curation, Formal analysis, Methodology, Project administration, Writing—review & editing. SE: Conceptualization, Formal analysis, Funding acquisition, Writing—review & editing. AAb: Conceptualization, Data curation, Formal analysis, Methodology, Writing—review & editing. AAl: Conceptualization, Formal analysis, Methodology, Writing—review & editing.
